# Pregnancy Outcome after Office Microhysteroscopy in Women with Unexplained Infertility

**DOI:** 10.22074/ijfs.2015.4237

**Published:** 2015-07-27

**Authors:** Emaduldin Mostafa Seyam, Momen Mohamed Hassan, Mohamed Tawfeek Mohamed Sayed Gad, Hazem Salah Mahmoud, Mostafa Gamal Ibrahim

**Affiliations:** 1 Department of Obstetrics and Gynecology, College of Medicine, El Minya University, Minya, Egypt; 2 Department of Obstetrics and Gynecology, Al Fayoum General Hospital, Fayoum, Egypt

**Keywords:** Abnormal Uterine Bleeding, Polyps, Fibroids, Cost-Effectiveness

## Abstract

**Background:**

Hysteroscopy offers diagnostic accuracy and the ability to treat uterine pathology. The current study aimed to review the findings and feasibility of the proposed office-based diagnostic and operative microhysteroscopy in previously diagnosed wom- en with unexplained infertility and to evaluate the post-microhysteroscopic pregnancy outcome in a-year follow-up period.

**Materials and Methods:**

This prospective controlled study was conducted between 2006 and 2013. Two hundreds women with unexplained infertility were randomized into two groups: A. study group including 100 women recruited for office micohysteroscopic session and B. control group including 100 without the proposed microhysteroscopic intervention. A malleable fiberoptic 2-mm, 0 and 30 degrees angled hysteroscopy along with an operative channel for grasping forceps, scissors, or coaxial bipolar electrode were used for both diagnostic and operative indications. The findings, complications, and patient tolerance were recorded. A-year follow-up of pregnancy outcome for both groups was also performed.

**Results:**

Seventy cases (70%) of patients had a normal uterine cavity. Twenty wom- en (20%) had endometrial polyps. Other pathology included submucous myomas in 3 cases (3%), intrauterine adhesions in 3 cases (3%), polypoid endometrium in 3 cases (3%), and bicornuate uterus in one case (1%). The pathological findings were treated in all patients without complication. Also a-year follow-up of the to- tal developing cumulative pregnancy rate (CPR) was evaluated in groups A and B (control). Group A revealed the total CPR of 28.5%, among which 25% in women without pathology, 40% in women with endometrial polyps, 23% in women with adhesions, 33% in women with polypoid endometrium, and 21% in those with bi- cornuate uterus. However, A-year follow-up of spontaneous pregnancy outcome in group B showed a total CPR of 15%.

**Conclusion:**

Women tolerance, safety, and feasibility of simultaneous operative correc- tion make the proposed office microhysteroscopy an ideal and routine procedure in order to diagnose and to treat missed intrauterine abnormalities, especially in cases with un- explained infertility, with additional improvement of the pregnancy outcome after the procedure.

## Introduction

Hysteroscopy is still considered the gold standard procedure for uterine cavity exploration. Hysteroscopy is only recommended by the World Health Organization (WHO) when clinical or complementary exams [ultrasound or hysterosalpingogram (HSG)] suggest intrauterine abnormality or after *in vitro* fertilization (IVF). However, many gynecologists feel that hysteroscopy is a more accurate tool because of the high false-positive and false-negative rates of intrauterine abnormality with HSG. Therefore, many specialists have used hysteroscopy as their first-line of routine exam for infertility patients regardless of guidelines (1-5).

Recently, Hystero-Salpingo-Contrast-Sonography (HyCoSy), saline infusion sonography (SIS) and gel infusion sonography (GIS) are inexpensive and non-invasive techniques, while they have been shown to be excellent diagnostic tools to detect subtle intrauterine abnormalities, but they are still so many missed diagnosis. Office hysteroscopy has been increasingly recommended as a routine procedure in the infertility work-up. It has become easy to perform in an outpatient setting without anesthesia. Moreover, it offers direct visualization and enables specialists to diagnose and to treat intrauterine pathology during the same office session (6-10).

Although hysteroscopy is generally accepted as the gold standard in diagnosis and treatment of uterine cavity pathology, many gynecologist are reluctant to perform hysteroscopy as an initial test without a high degree of suspicion for pathology due to the need for anesthesia in an operating room setting. Therefore, the advent of smaller diameter instruments makes office-based operative hysteroscopy as an ideal first-line procedure and can efficiently treat infertile patients with uterine abnormalities in the same setting, thus facilitating a rapid transition from diagnosis to treatment and subsequent pregnancy (11, 12).

The objective of this study was to review officebased diagnostic and operative microhysteroscopic findings and treatment in women with unexplained infertility to evaluate whether microhysteroscopy should be recommended to these patients who had the diagnosis of missed uterine abnormalities and to evaluate the impact of this proposed office procedure on subsequent pregnancy outcome for those women.

## Materials and Methods

Two hundreds infertile women, previously diagnosed as unexplained infertility, were recruited for the study between 2006 and 2013. The participants were randomized using a computer software into two groups: A. study group including 100 infertile women who were shortlisted for the studied office microhysteroscopic procedure and B. control group including 100 women with unexplained infertility who were followed up without the proposed office microhysteroscopic intervention. The demographic characters of the women are shown in table 1. The institutional ethical board approval was obtained for women in both groups recruited in Arafa Hospital (a private hospital) in Fayoum city. Each couple signed an appropriate informed consent for the procedure.

**Table 1:** Demographic characters of the women included in the study


Parameter	Cases (n=100)	Control (n=100)

Age (Y)	25± 5	26± 3
Menarche age (Y)	12.5 ± 2.5	11.1 ± 3
Regular cycles	89 ± 4	90 ± 3
Weight (kg)	60 ± 5	57 ± 4
Height (m)	1.57 ± 2.3	1.61 ± 1.6
BMI (kg/m2)	24 ± 3.6	23 ± 1.7
Type of infertility		
Primary	70	75
Secondary	30	25
Duration of infertility	2 ± 2.1	2.1 ± 1.3
Previous ART:		
IUI	40 cycles	38 cycles
ICSI	12 cycles	11 cycles


BMI; Body mass index, ART; Assisted reproductive techniques, IUI; Intra-uterine Insemination and ICSI; Intra-cytoplasmic sperm injection.

All office microhysteroscopies were performed using a malleable 0-degree diagnostic and 30-degrees operative 2-mm fiberoptic microhysteroscope (Circon, Germany) with an operative channel for the use of grasping forceps, scissors, or coaxial bipolar electrode. Instruments were placed through the built in operative channel when needed for treatment of pathology after the diagnostic portion had been completed. Typically, less than 1 liter of normal saline was used as the distention media for procedures, except with myomectomies which occasionally required larger volumes.

Operative procedures including hysteroscopic resection of endometrial polyps and submucous myomas, excision of intrauterine septum and postoperative management plan for bicornuate uterus were performed, where another conventional operative session for bicornuate uterus was arranged by another team. For those longer cases, fluid balance was monitored by ancillary staff throughout the procedure. Diagnostic findings, operative outcomes, complications, and patient tolerance during the procedure were noted.

The coaxial bipolar electrode surgical system (Versapoint, Gynecare, NJ) was used for myomectomies. Power settings were from 60 W (desiccation) to 130 W (cutting). Office microhysteroscopies were performed during the early postmenstrual period. Patients received oral premedication with midazolam (Sigma, Egypt), intramuscular analgesia with diclofenac (Epico, Egypt), and a paracervical uterine block with 1% lidocaine (Kahira, Egypt). Five patients requested conscious sedation with intravenous fentanyl (Cid, Egypt) and midazolam in place of the above regimen. All women were discharged immediately after the procedure, except those who were discharged after 2 hours due to prolonged operative indications.

All patients had a transvaginal ultrasound scanning performed in the office prior to the procedure to screen for uterine pathology, including uterine anomalies and intramural or subserosal myomas, as well as to assess uterine position. Those patients with an anteverted uterus had a full bladder at the time of microhysteroscopy to facilitate placement of the microhysteroscope.

Women tolerance during the whole procedure, pain perception scoring, the need for intraoperative conscious sedation, an extra postoperative analgesia, and the duration of the postoperative and lapse period before discharge were recorded to be analyzed (3).

For a 12-month follow-up period, pregnancy outcome were evaluated after the office microhysteroscopic procedure in A and B groups, for spontaneous pregnancy without any intervention, while each pregnancy developed after the microhysteroscopic procedure was correlated to each uterine abnormality diagnosed and treated during the microhysteroscopic procedure. Early pregnancy complications were evaluated for both groups, and some of the successful ongoing pregnancies were recorded as well.

### Statistical analysis

Chi-square test and students’ t test were used to analyze different sub-groups. Univariate and multivariate logistic regression were applied in order to identify factors that could predict the presence of unsuspected uterine cavity abnormalities. A P<0.05 was considered statistically significant. All statistical analyses were performed in SPSS version 15.1 (SPSS Inc., IL, USA).

## Results

**Fig.1:**
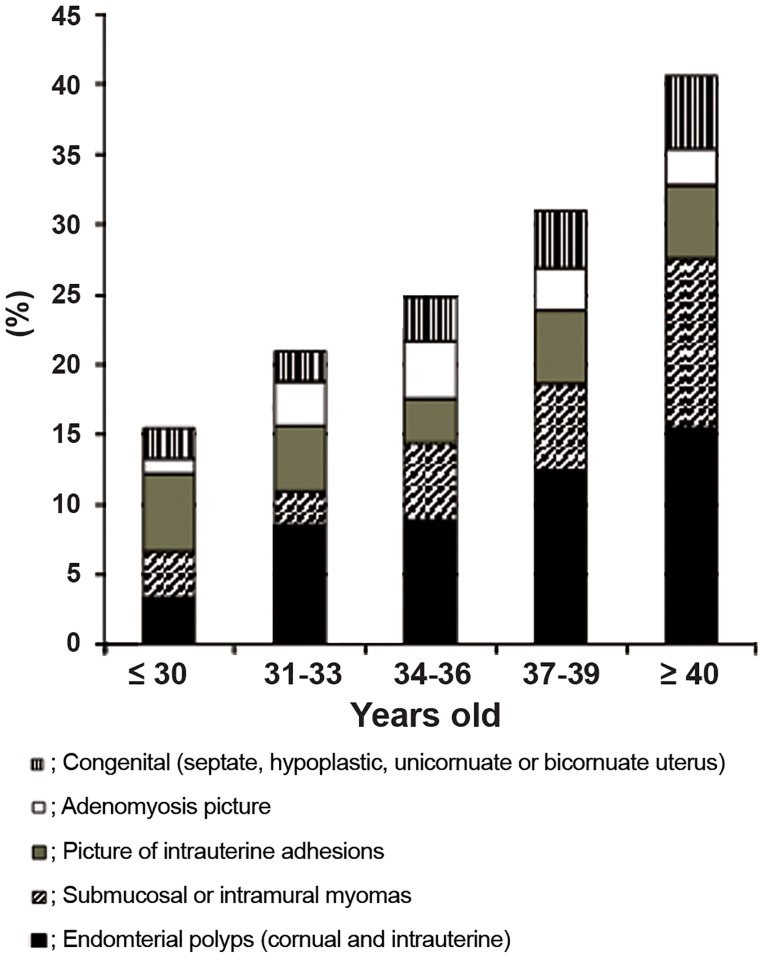
Microhysteroscopic abnormalities in relation to women age.

Table 1 shows the different demographic characteristics of the women included, indicating there are no significant differences between the case and control subjects. Table 2 lists the findings, both normal and pathologic cases, of the 100 office microhysteroscopies performed. Figure 1 shows the intrauterine abnormal findings in relation to women age category. All procedures were performed without complications. Treatment of adhesions and removal of polyps and submucous myomas were undertaken and completed in all patients. Division of septi was performed in patients with a known single fundus confirmed by laparoscopy at a prior time. No procedures were aborted secondary to patient intolerance.

Abnormalities included the followings: i. Atypical polypoid adenomyoma of endometrium in 3 cases (3%), ii. Intrauterin adhesion (IUA) synechiae in 3 cases (3% of all microhysteroscopies), iii. A case with uterus bicornis (1% of all microhysteroscopies), v. Submucous myoma in 3 cases (3% of all microhysteroscopies), vi. Deformed uterine cavity resulting from intramural myoma in 6 cases (6% of all microhysteroscopies), vii. Endometrial polyps in 20 cases (20% of all microhysteroscopies) and viii. unique in 10 cases (10% of all microhysteroscopies). Their location was either corporeal (14 cases) or cornual (6 cases). Table 3 shows the degree of patient compliance of the women included for the studied office microhysteroscopic procedure without general anesthesia. Most women accepted the procedure with a good degree of compliance, and none of the procedures was aborted due to the patient non-compliance.

The microhysteroscopic images were quite similar to those following the conventional 5-mm hysteroscopy, and might be better. Normal microhysteroscopic image appears as a regular cavity with reddish glistening endometrial lining with both ostial openings seen as black spots at 2 and 10 o’clock of the uterine cavity. Endometrial polyps and polypoid endometrium could be easily diagnosed, although mostly available together. Intrauterine adhesions could also diagnosed with a change of the reddish glistening soft endometrium to become rough non-glistening with areas of whitish myomas. Submucosal myomas could be diagnosed with the raised endometrial lining.

Table 2 shows the cumulative pregnancy rate during the postoperative one-year follow-up, following the office microhysteroscopic procedure, appears to be 25% in women without pathology (spontaneous pregnancies without interventions), 40% for endometrial polyps, 35% for adhesions, 33% for polypoid endometrium, and 22% for bicornuate uterus. The average total ongoing pregnancy rate is 25% after office microhysteroscopic procedure in group A versus 15% in group B. In group A, the best pregnancy rate belonged to after treated endometrial polyps and worst rate belong to the abnormal uterine configuration in uterus bicornis (0%). The total miscarriage rate is not significantly different in developing pregnancies after the different corrected abnormalities, managed after the office microhysteroscopic procedure. None of the office microhysteroscopic procedure was aborted.

**Table 2:** Office microhysteroscopic findings of 100 women with unexplained infertility and the reproductive outcomes after the procedure in group A compared to the related values in group B


Findings	Cases n (%)	CPR n (%)	OPR n (%)

Normal finding	70 (70 %)	35 (25%)	28 (20%)
Endometrial polyps	20 (20%)	16 (40%)	12 (30%)
Submucous fibroids	3 (3%)	2 (34%)	1 (23%)
Intrauterine adhesions	3 (3%)	1 (23%)	1 (22%)
Polypoid endometrium	3 (3%)	2 (33%)	1 (23%)
Bicornuate uterus	1 (1%)	1 (21%)	0 (0 %)
Total number in group A	100	57 (28.5%)	43 (21.5%)
Total number in group B	100	15 (15%)	10 (10%)


CPR; Cumulative pregnancy rate and OPR; Ongoing pregnancy rate.

**Table 3:** Patients compliance during and after the office microhysteroscopy


Patient compliance	n (%)

No or minor discomfort	90 (90%)
Discomfort	5 (5%)
Major discomfort	3 (3%)
Difficult examination	2 (2%)
Total	100


## Discussion

The basic infertility work-up has included a HSG to evaluate the uterine cavity and tubal patency. However, HSG does not allow for simultaneous correction of uterine pathology. Moreover HSG may miss 35% of uterine abnormalities. The high false-negative rate, the low-positive predictive value, and the inability to treat abnormal findings concurrently with the diagnosis have limited the use of HSG to assess the endometrial cavity (12-15).

Sonohysterography (SHG) has been proposed as a better diagnostic test of the uterine cavity. However, it also suffers from a sensitivity and specificity inferior to that of hysteroscopy in most studies. Additionally, it does not allow for correction of presumed pathology. Perhaps because hysteroscopy has traditionally required general anesthesia in an operating room setting, physicians do not consider hysteroscopy as a first-line test. Additionally, distention media are typically composed of low osmolality and electrolyte-free for operative work, and thus require careful surveillance of fluid status to minimize complications of hyponatremia and fluid overload. These requirements have made many practitioners reluctant to perform operative hysteroscopy (16-18).

Patient tolerance of hysteroscopes 2-5-mm allows for their use in an office setting where anesthesia is not required. Additionally, office hysteroscopy is no more costly than HSG at many institutions. Moreover using newly advanced microhysteroscope favors it over other tools. The professional fees for performing and reading a hysterosalpingogram (HSG) are 30% higher than the cost of an office hysteroscopy. Although SHG may offer a cost reduction, for many patients in whom pathology is found or suspected, a hysteroscopy is then indicated adding expense, delay, and inconvenience (18-22).

It has been reported that up to 20-50% of infertile patients have uterine abnormalities (30% in this study), including myomas, polyps, intrauterine adhesions, and uterine malformations. This is in agreement with our study that found 30% of patients undergoing office micohysteroscopy had uterine pathology. The high incidence of endometrial polyps in some patients may be related to prior therapy with gonadotropins due to higher levels of estrogen. Because pathology is present in 20 to 50% of infertile patients, as mentioned previously, practitioners should be more inclined to recommend hysteroscopy as part of the infertility work-up in conjunction with the routine laparoscopy and dye test, due to its simultaneous operative management (23-26).

Outpatient hysteroscopy has been shown to be easily performed with excellent surgical results in previous studies. Nagele et al. (26) and Vercellini et al. (27) found comparable success rates of 98% for performing the procedure. In this study, it was quite possible to perform all diagnostic and operative procedures in the office setting. Grasping forceps allow for removal of polyps with the ability to retain a clean specimen for pathologic confirmation. Scissors can be introduced for adhesions and septi. Bipolar electrode allows for removing submucous myomas. Using the cutting mode is primarily used for preservation of the delicate endometrium, minimizing the risk of postoperative adhesions, whereas the desiccation mode is applied when specific blood vessels are encountered.

Using saline as uterine distention medium helps to minimize medium-related complications. Hyponatremia and cerebral edema are of a concern when using hypotonic, electrolyte-free media, such as glycine or sorbitol. However, fluid overload, pulmonary edema, and congestive heart failure are likely to occur when an excessive volume of saline is used, especially if patients have underlying medical conditions predisposing them to fluid-related complications.

Air embolism is also considered as a potential complication. This could be minimized by avoiding to place the patients in an exaggerated Trendelenburg positioning, excessive fluid pressure overflow, prolonged operative times, dilating the cervix without instruments sealing air entry, and purging the tubing of air. Post-procedure complications like endometritis could be reduced by pre- and post-treatment with prophylactic antibiotics (16-18), and by avoiding operating on patients with known active vaginal infections (22-28). Patient tolerance of the electrosurgical equipment was excellent, confirming what El Toukhy et al. (29), Lorusso et al. (30) found in their studies on outpatient hysteroscopy.

Office-based operative hysteroscopy has also been found to be extremely safe. In this study, no complications occurred, and no patients needed extended monitoring or laboratory studies for fluid overload. Typical complications associated with hysteroscopy may be procedure-related, mediarelated, or post-procedure-related. Procedure-related complications, such as uterine perforation; cervical laceration; and damage to tissues including bowel, bladder, and vagina, could be almost minimized using the proposed office malleable 2-mm fiberoptic microhysteroscope, which did not need any cervical dilatation, passing smoothly within the undilated cervix. Moreover, the images produced were nearly similar or almost better than those after using the conventional 5-mm lens system hysteroscope, leading to minimal degree of patient’s discomfort.

An increase in pregnancy rates after performing office microhysteroscopic procedure might be attributed to the removal of endometrial polyps, polypoid endometrium, submucous myomas, or intrauterine synechiae at the time of microhysteroscopy that resulted in improving implantation in this population at risk. However, those pregnancies developed after microhysteroscopic confirmation of absence of any intrauterine pathology, the irrigation of the cavity with saline may have a beneficial effect on implantation and pregnancy rates in those women, as suggested in previous studies (20, 25, 30). The explanation of the highest pregnancy rate after excisions of polyps and myomas is logic, but the least pregnancy rate that was observed with uterus bicollis or acutely arcuate uterus might be due to the abnormal uterine cavity configuration. Suspected associated non-mechanical factors with diagnosed adenmyosis may explain the relatively lower pregnancy outcome developed after the procedure.

The higher ongoing pregnancy rate after the managed polyps, polypoid endomtrium, submucous myoma, and those after exclusion of any pathology might confirm the causality of those abnormalities as the main etiology for embryo implanatation, either mechanically or biochemical; however, after confirmation of the integrity of the endometrium and uterine wall, it is suggested to keep pregnancy safe beyond 20 weeks gestation. Regardless of whether these adjunctive benefits are confirmed by further study, office-based operative microhysteroscopy is definitely hold a great value as the gold standard of diagnostic procedures for uterine cavity abnormalities with the ease, safety, and efficiency of simultaneous therapeutic correction of abnormalities.

The spontaneous pregnancy outcome during the follow-up of group B was within the reported incidence before, although it was significantly lower than those following the microhysteroscopic procedure in group A. Taking into account, using any of the assisted reproductive techniques (ART) might increase this lower pregnancy outcome, but both groups were followed up without using any of those techniques, as that might interfere with the final pregnancy outcome. Still this spontaneous pregnancy outcome in group B was developed with no surgical intervention; no use of any type of anesthesia, conscious sedation, or analgesia; and no application of office procedure that was used for group A.

So our findings showed that in infertile population where office microhysteroscopy is performed routinely prior to the confirmation of un-explained cases of delayed conception, a significant percentage of patients are found to have uterine pathology, which had been missed to be diagnosed by the routine fertility work-up performed before. Endometrial polyps were found most frequently, with smaller numbers of myomas, adhesions, and septi. These abnormalities may impair the success of future treatment cycles, so removal of the pathology was advised. Patient tolerance and the feasibility of operative management, simultaneous with diagnosis, would make the proposed office-based operative microhysteroscopy in conjunction with/or after the routine laparoscopy as an ideal first-line procedure with minimal risk to the patient.

## Conclusion

Scheduling the office microhysteroscopy as one of the routine steps in the fertility work-up program has become mandatory before the final diagnosis of unexplained infertility. This technique is considered not only an ideal gold test to diagnose many intrauterine abnormalities that are undiagnosed with other routine tools, but also the significant improvement in the pregnancy outcome following the microhysteroscopic procedure, supports the previously mentioned recommendation. In addition, it is recommended to conduct future research works to support this recommendation.

## References

[1] Frydman R, Eibschitz I, Fernandez H, Hamou J (1987). Uterine evaluation by microhysteroscopy in IVF candidates. Hum Reprod.

[2] Shokeir TA, Shalan HM, El-Shafei MM (2004). Combined diagnostic approach of laparoscopy and hysteroscopy in the evaluation of female infertility: results of 612 patients. J Obstet Gynaecol Res.

[3] El-Mazny A, Abou-Salem N, El-Sherbiny W, Saber W (2011). Outpatient hysteroscopy: a routine investigation before assisted reproductive techniques?. Fertil Steril.

[4] Mooney SB, Milki AA (2003). Effect of hysteroscopy performed in the cycle preceding controlled ovarian hyperstimulation on the outcome of *in vitro* fertilization. Fertil Steril.

[5] Kupesic S, Kurjak A, Skenderovic S, Bjelos D (2002). Screening for uterine abnormalities by three-dimensional ultrasound improves perinatal outcome. J Perinat Med.

[6] Munro MG (2010). Complications of hysteroscopic and uterine resectoscopic surgery. Obstet Gynecol Clin North Am.

[7] De Sa Rosa e de Silva AC, Rosa e Silva JC, Candido dos Reis FJ, Nogueira AA, Ferriani RA (2005). Routine office hysteroscopy in the investigation of infertile couples before assisted reproduction. J Reprod Med.

[8] Vilos GA (1999). Intrauterine surgery using a new coaxial bipolar electrode in normal saline solution (Versapoint): a pilot study. Fertil Steril.

[9] Fernandez H, Gervaise A, De Tayrac R (2000). Operative hysteroscopy for infertility using normal saline solution and a coaxial bipolar electrode: a pilot study. Hum Reprod.

[10] Guida M, Pellicano M, Zullo F, Acunzo G, Lavitola G, Palomba S (2003). Outpatient operative hysteroscopy with bipolar electrode: a prospective multicentre randomized study between local anaesthesia and conscious sedation. Hum Reprod.

[11] Cooper NA, Smith P, Khan KS, Clark TJ (2011). A systematic review of the effect of the distension medium on pain during outpatient hysteroscopy. Fertil Steril.

[12] Molinas CR, Campo R (2006). Office hysteroscopy and adenomyosis. Best Pract Res Clin Obstet Gynaecol.

[13] Preutthipan S, Linasmita V (2003). A prospective comparative study between hysterosalpingography and hysteroscopy in the detection of intrauterine pathology in patients with infertility. J Obstet Gynaecol Res.

[14] Pansky M, Feingold M, Sagi R, Herman A, Schneider D, Halperin R (2006). Diagnostic hysteroscopy as a primary tool in a basic infertility workup. JSLS.

[15] Silberstein T, Saphier O, vanVoorhis BJ, Plosker SM (2006). Endometrial polyps in reproductive-age fertile and infertile women. Isr Med Assoc J.

[16] Cicinelli E, Resta L, Nicoletti R, Zappimbulso V, Tartagni M, Saliani N (2005). Endometrial micropolyps at fluid hysteroscopy suggest the existence of chronic endometritis. Hum Reprod.

[17] Kowalczyk D, Guzikowski W, Więcek J, Sioma-Markowska U (2012). Clinical value of real time 3D sonohysterography and 2D sonohysterography in comparison to hysteroscopy with subsequent histopathological examination in perimenopausal women with abnormal uterine bleeding. Neuro Endocrinol Lett.

[18] Shokeir A, Shalan HM, El-Shafei MM (2004). Combined diagnostic approach of laparoscopy and hysteroscopy in the evaluation of female infertility: results of 612 patients. J Obstet Gynaecol Res.

[19] Siristatidis C, Chrelias C, Salamalekis G, Kassanos D (2010). Office hysteroscopy: current trends and potential applications: a critical review. Arch Gynecol Obstet.

[20] Van Dongen H, De Kroon CD, Jacobi CE, Trimbos JB, Jansen FW (2007). Diagnostic hysteroscopy in abnormal uterine bleeding: a systematic review and meta-analysis. BJOG.

[21] Koskas M, Mergui JL, Yazbeck C, Uzan S, Nizard J (2010). Office hysteroscopy for infertility: a series of 557 consecutive cases. Obstet Gynecol Int.

[22] Bettocchi S, Nappi L, Ceci O, Selvaggi L (2004). Office hysteroscopy. Obstet Gynecol Clin North Am.

[23] Bozdag G, Aksan G, Esinler I, Yarali H (2008). What is the role of office hysteroscopy in women with failed IVF cycles?. Reprod Biomed Online.

[24] Cohen MJ, Rosenzweig TS, Revel A (2007). Uterine abnormalities and embryo implantation: clinical opinion altered by peer debate. Reprod Biomed Online.

[25] Demirol A, Gurgan T (2004). Effect of treatment of intrauterine pathologies with office hysteroscopy in patients with recurrent IVF failure. Reprod Biomed Online.

[26] Nagele F, O'Connor H, Davies A, Badawy A, Mohamed H, Magos A (1996). 2500 Outpatient diagnostic hysteroscopies. Obstet Gynecol.

[27] Vercellini P, Cortesi I, Oldani S, Moschetta M, De Giorgi O, Crosignani PG (1997). The role of transvaginal ultrasonography and outpatient diagnostic hysteroscopy in the evaluation of patients with menorrhagia. Hum Reprod.

[28] Doldi N, Persico P, Di Sebastiano F, Marsiglio E, De Santis L, Rabellotti E (2005). Pathologic findings in hysteroscopy before *in vitro* fertilization-embryo transfer(IVF-ET). Gynecol Endocrinol.

[29] El-Toukhy T, Campo R, Sunkara SK, Khalaf Y, Coomarasamy A (2009). A multi-centrer randomised controlled study of pre-IVF outpatient hysteroscopy in women with recurrent IVF implantation failure: Trial of Outpatient Hysteroscopy -[TROPHY] in IVF. Reprod Health.

[30] Lorusso F, Ceci O, Bettocchi S, Lamanna G, Constantino A, Serrati G, Depalo R (2008). Office hysteroscopy in an in vitro fertilization program. Gynecol Endocrinol.

